# Chern structure in the Bose-insulating phase of Sr_2_RuO_4_ nanofilms

**DOI:** 10.1038/srep41291

**Published:** 2017-01-23

**Authors:** Hiroyoshi Nobukane, Toyoki Matsuyama, Satoshi Tanda

**Affiliations:** 1 Department of Physics, Hokkaido University, Sapporo, 060-0810, Japan; 2 Center of Education and Research for Topological Science and Technology, Hokkaido University, Sapporo, 060-8628, Japan; 3 Department of Physics, Nara University of Education, Nara 630-8528, Japan; 4 Department of Applied Physics, Hokkaido University, Sapporo 060-8628, Japan

## Abstract

The quantum anomaly that breaks the symmetry, for example the parity and the chirality, in the quantization leads to a physical quantity with a topological Chern invariant. We report the observation of a Chern structure in the Bose-insulating phase of Sr_2_RuO_4_ nanofilms by employing electric transport. We observed the superconductor-to-insulator transition by reducing the thickness of Sr_2_RuO_4_ single crystals. The appearance of a gap structure in the insulating phase implies local superconductivity. Fractional quantized conductance was observed without an external magnetic field. We found an anomalous induced voltage with temperature and thickness dependence, and the induced voltage exhibited switching behavior when we applied a magnetic field. We suggest that there was fractional magnetic-field-induced electric polarization in the interlayer. These anomalous results are related to topological invariance. The fractional axion angle Θ = *π*/6 was determined by observing the topological magneto-electric effect in the Bose-insulating phase of Sr_2_RuO_4_ nanofilms.

The mathematical structure characterized by Chern numbers^1^ has yielded very important findings in both condensed-matter and high-energy physics^2^. The quantum Hall effect in graphene provides the quantized Hall conductance of 
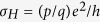
 with *p* and *q* coprimes^3^. The quantization of the Hall current and anyonic particles with exotic mutual statistics can be explained by the Chern-Simons (CS) term in (2 + 1)-dimensional topological field theory, which is known as the parity anomaly^4^,^5^,^6^. In 3 + 1 dimensions, the neutral pion decay^7^,^8^ and the creation of excitation momentum by quantized vortices in the superfluid ^3^He^9^ are described by the chiral anomaly. However, as yet there has been no experimental evidence of quantum anomalies and Chern structures in superconductors. Recently, the magneto-electric effect in topological superconductors and insulators has been predicted theoretically from the Chern-Pontryagin term (topological Θ-term with Θ = ±*π*) in 3 + 1 dimensions^10^,^11^. The Chern structure in superconductivity is interesting in itself and may also have implications regarding the esoteric physics of quantum chromodynamics (axion electrodynamics)^12^,^13^ in condensed-matter experiments. In addition to the purely scientific interest that it arouses, the Chern structure may provide the basis for new applications to magneto-electric coupling devices and for the development of the topological quantum computation of non-Abelian statistics^14^.

Layered perovskite Sr_2_RuO_4_ is a leading candidate for a spin-triplet and chiral *p*-wave superconductor in quasi-two-dimensional electron systems^15^, and is also known as a Chern superconductor with a non-zero Chern invariant. The spontaneously broken time-reversal and parity symmetry realize novel topological quantum phenomena such as zero-magnetic-field quantum Hall effects^16^,^17^,^18^, gapless Majorana excitations in an edge or the core of vortices^19^ and the non-Abelian statistics of half-quantum vortices^20^. However, the chiral-multi-domains in millimeter-scale Sr_2_RuO_4_ obscure these novel phenomena. We have reported that the current-voltage (*I*–*V*) curves in the microscale chiral single domain of Sr_2_RuO_4_ with submicron thickness violate parity due to the excitation of the Majorana-Wyle fermions along the one-dimensional chiral edge current^21^,^22^. To clarify the Chern structure through quantum transport in units of *e*^2^/*h* in two-(or quasi-two-) dimensional chiral superconducting layers, we have investigated electric transport properties by using chiral single domain size Sr_2_RuO_4_ with nanoscale thickness^23^. Specifically, in this paper, we report the fractionalized Chern structure (number) in the quantum critical region from the results of the anomalous properties, which are revealed by reducing the Sr_2_RuO_4_ thickness to the nanometer range.

One unsolved problem in Sr_2_RuO_4_ systems is the two superconducting phases with *T*_*c*_ ~ 1.5 and 3 K. Although pure Sr_2_RuO_4_ single crystals exhibit a *T*_*c*_ of about 1.5 K, enhancement to about 3 K has been reported in Sr_2_RuO_4_-Ru eutectic systems^24^. However, recent investigations have found that, even in pure Sr_2_RuO_4_ without Ru inclusions, an enhanced *T*_*c*_ of around 3 K is observed when measuring uniaxial pressure effects along the *c* axis^25^, strain effects^26^ and the properties near the lattice dislocations^27^. As regards this discrepancy, electric transport measurements in nanoscale thin films of Sr_2_RuO_4_ single crystals allow access to both topological quantum states and the pairing mechanism itself in chiral *p*-wave superconductors.

In this paper, we report the emergence of a Chern structure in the Bose-insulating phase of Sr_2_RuO_4_ single crystal nanofilms based on the anomalous transport properties observed for the in-plane and interlayer directions. By reducing the Sr_2_RuO_4_ thickness to the nanometer range, we found that a fractional quantum Hall resistance of *h*/4*e*^2^ − *h*/2*e*^2^ occurred as a consequence of the spontaneous Hall current without an external magnetic field. The gap structure below 3 K shows localized superconducting islands connected by tunnel junctions. The anomalous induced voltage and the switching behavior were observed as a function of temperature and thickness under zero bias current for an applied magnetic field parallel to the *c* axis. The applied magnetic field induced electric polarization in the interlayer of Sr_2_RuO_4_ nanofilms. In Sr_2_RuO_4_ with a nanoscale thickness, we suggested that the fractional topological magneto-electric effect occurs in three (quasi-two) dimensions, which is characterized by the fractional axion angle (coefficient) Θ = *π*/6 of the ***E*** · ***B*** term in the chiral anomaly.

## Results

**Figure 1:**
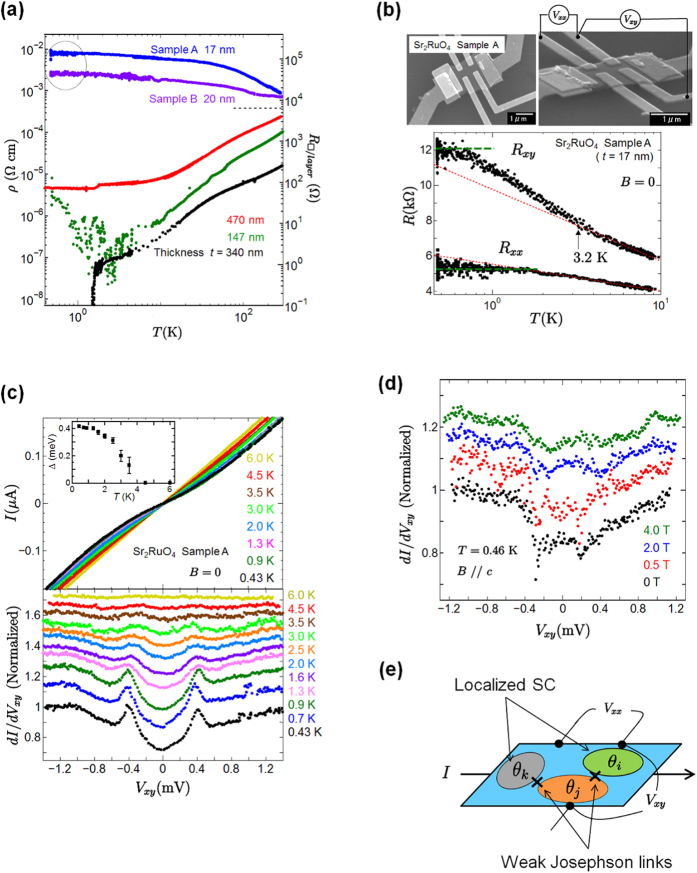
Superconductor-insulator transition driven by varying thickness and local superconductivity in Sr_2_RuO_4_ nanofilms. (**a**) Temperature dependence of the resistivity *ρ*_*xx*_ for different thicknesses of Sr_2_RuO_4_ single crystals. 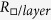 is resistance per square per RuO_2_ layer. The dotted horizontal line represents (*h*/4*e*^2^) = 6.45 kΩ. (**b**) Scanning electron micrographs of the top and side views of sample A. Temperature dependence of *R*_*xy*_ and *R*_*xx*_. The dotted horizontal lines are a guide for the eye. (**c**) *V*_*xy*_ − *I* characteristics for sample A at temperatures in a zero magnetic field. *dI/dV*_*xy*_ as a function of *V*_*xy*_. The inset shows the temperature dependence of the superconducting gap Δ. (**d**) Tunneling spectra *dI/dV*_*xy*_ for sample A in various magnetic fields. (**e**) Schematic of local superconducting islands weakly coupled by tunneling junctions, where *θ* is the superconducting phase. This may be similar to small Josephson junction arrays.

Figure 1(a) shows the temperature dependence of the longitudinal resistivity *ρ*_*xx*_ for various thicknesses of exfoliated Sr_2_RuO_4_ films. 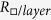 is resistance per square per RuO_2_. In general, bulk (thick) superconductors exhibit zero longitudinal resistivity below *T*_*c*_. We have observed zero resistivity below *T*_*c*_ = 1.59 K for microscale Sr_2_RuO_4_ with a thickness of 340 nm, which is consistent with the *T*_*c*_ of bulk Sr_2_RuO_4_ crystals^15^,^24^. Samples with a thickness of 147 and 470 nm exhibited a slight drop in resistivity around 1.5 ~ 3 K, and showed non-zero resistivity below *T*_*c*_. The result shows that the flow of vortices can be caused by quantum fluctuations of the superconducting phase^28^,^29^. Interestingly, the insulating behavior was observed in samples A and B with nanoscale thickness. It has been reported that the effect of a negative pressure acts on thin films as the thickness of the exfoliated films decreases to the nanometer range^30^. The pressure plays a key role in modifying the electric properties of the family of ruthenium oxides^31^. Thus we presume that the transition from superconductor to insulator appeared in Sr_2_RuO_4_ nanofilms as a result of reducing sample thickness to the nanometer range.

The two-dimensional superconductor-insulator transition allows a quantum resistance near the quantum critical point due to superconducting phase fluctuations^32^,^33^,^34^. Figure 1(b) shows the temperature dependence of the Hall resistance *R*_*xy*_ and longitudinal resistance *R*_*xx*_ in sample A with a thickness of 17 nm. With decreasing temperature, the Hall resistance increased with a log *T* dependence, and reached about 12.1 kΩ below 0.8 K. Interestingly, in the absence of an external magnetic field, we measured a Hall resistance of 12.1 kΩ, which is close to the quantum resistance of *h*/2*e*^2^. A Hall resistance of *R*_*xy*_ = 6.8 kΩ ~ *h*/4*e*^2^ was also observed in sample B (see Supplementary Fig. S1). The quantum Hall resistance was reproduced in other Hall electrodes and samples in the order of 1 ~ 20 kΩ. We note that the *R*_*xx*_ values of samples A and B were 5.3 kΩ ~ *h*/5*e*^2^ and 6.1 kΩ ~ *h*/4*e*^2^, respectively, at lower temperature. These values are very close to the universal resistances of 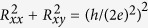 in a two-dimensional superconductor-insulator transition^35^,^36^. The universal resistance requires the existence of both localized Cooper pairs and moving vortices on the insulating side of the quantum phase transition, which is known as a Bose insulator. Thus we think that the Hall and longitudinal quantum resistance in Sr_2_RuO_4_ nanofilms is related to the dynamics of Cooper pairs and vortices in the Bose-insulating phase of two dimensions. The *R*_*xy*_ and *R*_*xx*_ values in the thick samples with thicknesses of a few hundred nanometers exhibited much smaller values of 0.1 ~ 1Ω than the quantum resistance in the thin film samples. We need to determine exact thickness dependence of quantized values of two dimensional samples and thick samples in future works.

We investigated the superconducting properties in the insulating phase of Sr_2_RuO_4_ nanofilms at low temperature. Figure 1(c) shows the *I*–*V*_*xy*_ characteristics of the Hall bar geometry, and *dI/dV*_*xy*_ as a function of the Hall voltage *V*_*xy*_ in a zero magnetic field at several temperatures, which is vertically shifted for clarity (see Supplementary Fig. S2 for *dI/dV*_*xx*_ − *V*_*xx*_). Surprisingly, below 3 K, clear gap structures were observed in both the Hall and the longitudinal conductance spectra. The temperature dependence of the superconducting gap extracted from the coherence peak width in the tunneling spectra is shown in the inset of Fig. 1(c). The result is comparable to the superconducting gap size found in previous reports on tunneling spectroscopy in Sr_2_RuO_4_ systems^37^,^38^,^39^,^40^,^41^. The appearance of the gap structure above *T*_*c*_ = 1.5 K reminds us of the pseudo-gap state in cuprate superconductors and the 3 K phase in Sr_2_RuO_4_ systems^24^,^25^,^26^,^27^. The result shows that the local superconducting islands connected by small Josephson junctions can emerge even in the insulating phase of Sr_2_RuO_4_ nanofilms below 3 K due to the quantum fluctuation of the superconducting phase *θ* as shown in Fig. 1(e). Thus, *V*_*xy*_ as well as *V*_*xx*_ is dependent on the current. The field dependence of the tunneling spectra for sample A at 0.46 K is shown in Fig. 1(d). The observation of the gap behavior at 4 T provides evidence for local superconductivity surviving up to high fields. Here a 400-nm-thick Sr_2_RuO_4_ single crystal (sample C) shows neither the suppression of *T*_*c*_ nor an enhancement to 3 K (see Supplementary Fig. S3).

**Figure 2:**
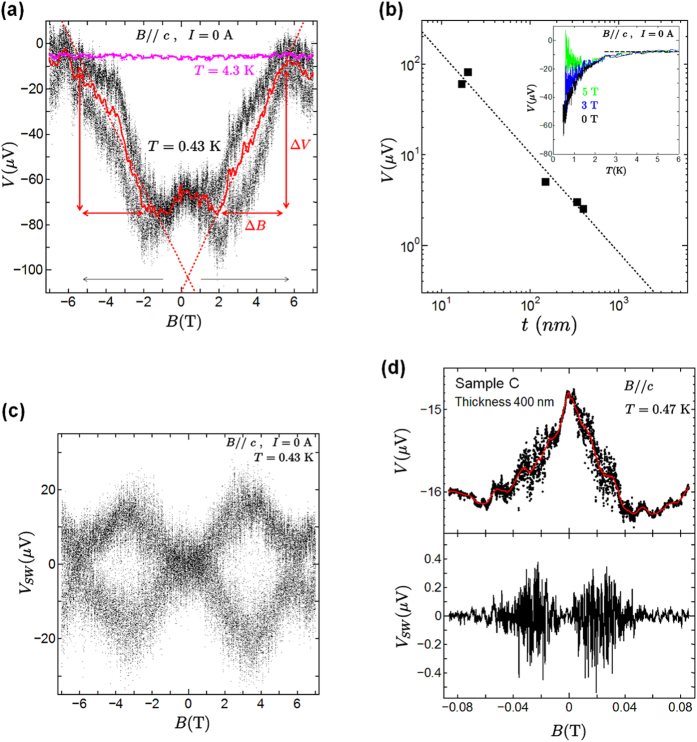
Topological magnetic-field-induced electric polarization. (**a**) Dependence of induced voltage on magnetic field at 0.43 and 4.3 K with zero bias current. Arrows represent the magnetic sweep direction from zero magnetic field to ±7 T. The solid red curve represents the average result for the measured data. (**b**) Thickness dependence of induced voltage at lower temperature. The dotted line represents the fitting result, which is described well by *V* = 1/*t*. The inset shows the temperature dependence of the induced voltage. (**c**) The anomalous switching voltage *V*_*SW*_ extracted from the *B* − *V* curves in (**a**). (**d**) Magnetic field dependence of *V* and *V*_*SW*_ for sample C.

**Figure 3:**
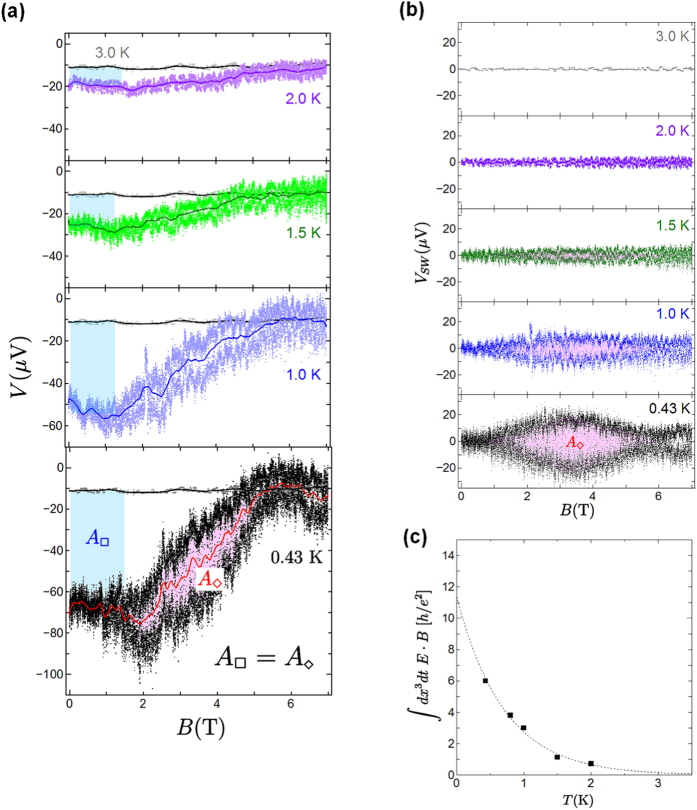
Temperature dependence of *E* · *B* value. (**a**) Magnetic field dependence of the anomalous voltage at various temperatures with zero bias current from zero magnetic field to 7 T. The *E* · *B* value in a low magnetic field (the blue region 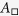) is equivalent to that in the red region 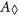 caused by voltage switching at temperatures below 2 K. (**b**) Temperature dependence of *V*_*SW*_. (**c**) Temperature dependence of the *E* · *B* integral value. The data were fitted by an exponential curve.

Quantum fluctuations near a quantum critical region bring out the topological properties of systems. To examine the topological magneto-electric coupling in the Bose-insulating phase of Sr_2_RuO_4_ nanofilms, we measured the voltage *V* with Hall-bar geometry and the longitudinal voltage *V*′ at zero bias current. Figure 2(a) shows the magnetic field dependence of the voltage *V* when a magnetic field is applied parallel to the *c* axis from a zero magnetic field up to ±7 T for sample A. Interestingly, we found an anomalous induced voltage of about |Δ*V*| = 62 V in a zero magnetic field at 0.43 K. In the longitudinal geometry, there was an induced voltage *V*′ of |Δ*V*′| = 40 *μ*V. The spontaneous voltage was reproduced in different terminals. In the Bose-insulating (local superconducting) state below 3 K, the anomalous voltage appeared as shown in the inset of Fig. 2(b), which is consistent with the *V* − *B* characteristics in Fig. 2(a). Figure 2(b) shows the thickness dependence of the induced voltage at lower temperature. As the sample thickness *t* was reduced, the induced voltage increased, which is fitted well by the relation *V* = 1/*t*. Since spontaneous voltage is related to sample thickness, we can eliminate the contribution of the thermoelectric voltage and a junction at the Au/Sr_2_RuO_4_ interface or at a microcrack. Furthermore, the switching behavior of an induced voltage was observed in the region between ±1.0 and ±5.8 T. The solid red line in Fig. 2(a) is the average result for the *B* − *V* characteristics. Figure 3(a) shows the magnetic field dependence of the induced voltage and the switching voltage at various temperatures. With increasing temperature, the anomalous induced voltage and the switching voltage were gradually suppressed, and vanished above 3 K. In sample C with a thickness of 400 nm, we also observed an induced voltage of 1 *μ*V and switching behavior under an applied magnetic field as shown in Fig. 2(d). The anomalies became smaller than the results observed for sample A. Table 1 summarizes the properties obtained by varying the thickness.

## Discussions

To analyze the switching phenomena in more detail, we subtract the average *B* − *V* curves from the measured *B* − *V* characteristics. The results for sample A are shown in Figs 2(c) and 3(b). Here the voltage *V*_*SW*_ represents the switching voltage component of the induced voltage. As the magnetic field increases, the anomalous switching voltage *V*_*SW*_ increases above ~±1 T. The *V*_*SW*_ for the applied magnetic field reaches its maximum value near ±3.5 T, and then decreases. Intriguingly, the switching voltage was clearly observed below 1.5 K. We think that the observation of this anomalous switching voltage is related to the intrinsic properties of the chiral *p*-wave superconductor Sr_2_RuO_4_, because the feature appears below a *T*_*c*_ of about 1.5 K in bulk Sr_2_RuO_4_.

We discuss the enhancement of the critical magnetic field in relation to the existence of localized superconducting islands of Sr_2_RuO_4_. In sample C as shown in Fig. 2(d), the magnetic field of 0.04 T caused the anomalous *V* and *V*_*SW*_ to vanish, which is consistent with *μ*_0_*H*_*c*2_ in bulk Sr_2_RuO_4_. On the other hand, in Figs 2(a) and 3(a), the anomalous voltage is induced even in a magnetic field beyond *μ*_0_*H*_*c*2_ reported for pure bulk Sr_2_RuO_4_. To reveal whether or not this anomalous behavior is an intrinsic characteristic of Sr_2_RuO_4_, we need to consider the physical properties of the 3 K phase. The 3 K superconductivity in Sr_2_RuO_4_-Ru systems induces the enhancement of the upper critical field to 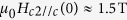 and 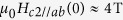
15. In general, the critical magnetic field in mesoscopic superconductors becomes larger than that in bulk superconductors. We estimated 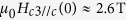 and 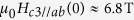 using *H*_*c*3_ = 1.7 *H*_*c*2_^42^. This may be comparable to the result for the *B* − *V* characteristics in our nanofilms because we observed the gap structure in a high field of 4 T as shown in Fig. 1(d).

Now let us consider the switching behavior of the resistivity (voltage) in superconducting systems. The Hall resistivity at very low temperature exhibited erratic switching in the vicinity of the quantum superconductor-to-insulator transition in La_2−*x*_Sr_*x*_CuO_4_, which is considered an indication of the charge-cluster glass state^43^. In the superconducting state of Sr_2_RuO_4_, the dynamics of the chiral domains generates switching behaviors as a function of magnetic field or time^44^. Our anomalous switching may resemble these results because the domains in the Bose-insulating phase of Sr_2_RuO_4_ fluctuate in space and time. At present, some possible explanations for the results are conceivable. Below, we discuss in detail a topological interpretation in terms of our results as an interesting possibility.

To understand the origin of the anomalous behaviors, we address (I) the fractional quantum Hall conductance in the conducting layer without a magnetic field, (II) the fractional magnetic-induced electric polarization in the interlayer, and (III) the integral value of *E* · *B* in the topological term. First, let us discuss the fractional quantum Hall conductance in a zero magnetic field in Sr_2_RuO_4_ nanofilms. From *R*_*xy*_ = 12.1 kΩ and *R*_*xx*_ = 5.3 kΩ for sample A, the sheet Hall conductance 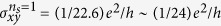 per RuO_2_ layer was determined by using the relation 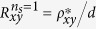, 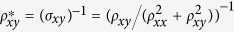, *d* = 6 Å (=*c*/2). By considering the number of sheets *n*_*s*_, we represent the Hall conductance as 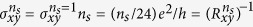. Why do Sr_2_RuO_4_ nanofilms exhibit Hall conductance quantized in the unit of conductance quantum 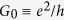
? The concept of chiral *p*-wave superconductivity can be developed by the induced CS-term of the effective Lagrangian in topological field theory^16^,^17^. The CS-term induces the existence of a spontaneous Hall current in a zero magnetic field perpendicular to the bias current direction. However, the quantum Hall effect in Sr_2_RuO_4_ has yet to be observed experimentally for the following reasons. The Hall resistance in a thick sample becomes smaller than that in a thin film sample, and the observation of the ensemble averaging of the Hall current in multi-chiral domains is complicated. With respect to these issues, an important solution is for the sample to consist of nanoscale thin films of ~10 layers^2^,^18^ and for the chiral single domain size to be ~1 *μ*m^21^. Our samples satisfy these conditions. Thus, by using Sr_2_RuO_4_ nanofilms, we can observe the fractional quantum Hall conductance near the quantum critical region.

Using the result shown by the fitted slope of |Δ*V*|/Δ*B* in Fig. 2(a), we discuss the contribution of the electric polarization under a magnetic field in the Sr_2_RuO_4_ interlayer. We assume that our layered sample is a superconducting bilayer system in order to discuss the possibility of magneto-electric polarization. The capacitance *C* = *ε*_*I*_*A/d* = 10 fF of the interlayer is estimated, where *ε*_*I*_/*ε*_0_ ~ 10 is the interlayer dielectric constant, *d* (=6 Å) is the interlayer distance in Sr_2_RuO_2_, and *A* is the area 0.14 *μ*m^2^ between the electrodes. We determined the effective electric charge *Q*^∗^ ~ 8 *e* from the induced voltage of |Δ*V*| = 63 *μ*V. Moreover, we obtained the effective magnetic flux 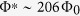 from the relation 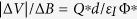, where 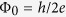 is the magnetic flux quantum. We found the fractional magneto-electric polarization 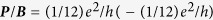 from the slope in the positive (negative) magnetic field. Surprisingly, the fractional coefficient of the magnetic-field-induced electric polarization is equivalent to that of the Hall conductance in the bilayer film.

Below, we consider the topological magneto-electric effect in the Bose-insulating phase of Sr_2_RuO_4_ nanofilms to understand the relationship between fractional Hall conductance and electric polarization. The chiral anomaly^7^,^8^ in the (3 + 1)-dimensional topological field theory can introduce an additional Θ-term 
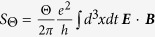
10,11. This topological term denotes the existence of the magneto-electric effects. Namely, an applied electric field generates a magnetic polarization 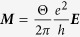, and an applied magnetic field generates an electric polarization 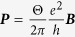. The quantum Hall current 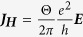 flows as the contribution of the topological surface state in the (3 + 1)-dimensional magneto-electric effect. For strongly correlated electron systems, the fractional parameter Θ = *p/q* with *p, q* odd integers is predicted by analogy with fractional quantum Hall effects^45^,^46^. This model of fractionalization in the chiral anomaly appears to be beneficial in terms of understanding our anomalous results.

Furthermore, we discuss the possibility of the fractionalization of the topological Θ-parameter. Using the experimental results, we discuss the integral value 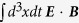 in the topological Θ-term. We found that the value of *E* (=Δ*V/d*) · *B* represented by the blue square region 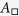 in Fig. 3(a) is equivalent to that of *E* · *B* represented by the red rhombic region 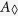 in Fig. 3(a,b). An important point is that the obtained value of *E* · *B* is 6(*h/e*^2^) at 0.43 K. Similarly, at temperatures below 2.0 K, we estimated the value of *E* · *B* in both the square region in a low magnetic field and the rhombic region provided by the voltage switching. We confirmed that the *E* · *B* values are the same in the blue and red regions at each temperature. The correspondence of the *E* · *B* value is also reproduced in sample C in Fig. 2(d). This means that the induced voltage in a low magnetic field is closely related to the occurrence of the switching voltage under a magnetic field, and these are connected to the topological invariant. Figure 3(c) shows the temperature dependence of the *E* · *B* value. The data points are fitted by an exponential curve. We believe that the *E* · *B* value exists in the 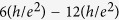 region. According to the fitting curve, at *T* = 0, (III) the *E* · *B* value is about 12 *h/e*^2^, which is comparable to (I) the zero-magnetic-field quantum Hall conductance 
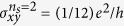
 and (II) the magnetic-field-induced electro-polarization 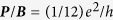. By substituting 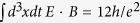 into the topological term *S*_Θ_, we obtained the fractional angle 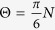, where *N* is an integer multiple. For the fractional angle Θ = *π*/6, the quantum Hall conductance 
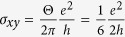
, and electric polarization 
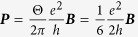
 are discussed theoretically^10^,^46^. This is consistent with our experimental observations. Thus, we suggested the presence of the fractional topological magneto-electric effect in Sr_2_RuO_4_ layers using the results of both the fractional Hall conductance and the fractional magneto-electric polarization in the fractional axion angle. Namely, these observations correspond to the fractional Chern structure caused by the quantum anomaly in Sr_2_RuO_4_.

In conclusion, we have detected the emergence of the Chern structure in Bose-insulating Sr_2_RuO_4_ nanofilms by observing the fractional Hall conductance on the surface and the fractional electric polarization in the interlayer. In a zero magnetic field, a quantized fractional Hall resistance was observed in the local superconducting state below 3 K. Under zero bias current, we found the anomalous induced voltage and the switching behavior of the induced voltage for an applied magnetic field parallel to the *c* axis. The applied magnetic field generated electric polarization in the interlayer of Sr_2_RuO_4_. The results suggest the presence of the fractional topological magneto-electric effect in Sr_2_RuO_4_ nanofilms. The fractional axion angle Θ = *π*/6 in the topological Θ-term was also determined.

## Methods

To obtain nanoscale Sr_2_RuO_4_ thin films, we synthesized Sr_2_RuO_4_ single crystals with a solid phase reaction, and selected single crystals with no embedded Ru metal and with homogeneity by observing optical microscope images, chemical composition and crystal orientation^47^. Sr_2_RuO_4_ single crystal nanofilms were exfoliated on a SiO_2_(300 nm)/Si substrate. We then fabricated gold electrodes using standard electron beam lithography methods. Scanning electron micrographs of the Sr_2_RuO_4_ nanofilms (samples A and B) are shown in Fig. 1(b) and Fig. S1(a). The sample thickness was determined from scanning electron micrographs obtained with a sample holder tilted at 70-degrees. The electric transport properties of several samples with thicknesses of 17–470 nm were measured by the four-terminal method using a homemade ^3^He refrigerator. All leads were equipped with *RC* filters (*R* = 1 kΩ and *C* = 22 nF). The longitudinal and Hall voltages and the differential conductance were measured with a nanovoltmeter (2182, Keithley) and a lock-in-amplifier (5210, Princeton Applied Research), respectively. The up and down magnetic field sweep rate was 0.102 mT/sec.

## Additional Information

**How to cite this article**: Nobukane, H. *et al*. Chern structure in the Bose-insulating phase of Sr_2_RuO_4_ nanofilms. *Sci. Rep.* **7**, 41291; doi: 10.1038/srep41291 (2017).

**Publisher's note:** Springer Nature remains neutral with regard to jurisdictional claims in published maps and institutional affiliations.

## Supplementary Material

Supplementary Information

## Figures and Tables

**Table 1:** Summary of the properties of Sr_2_RuO_4_.

	Sample A	Sample C
Thickness (nm)	17	400
*T*_*c*_ (K)	3	1.5
*R*_*xy*_ (kΩ)	12	1 × 10^−3^
Induced *V (μ*V)	62	1
*B* at maximum *V*_*SW*_ (T)	3.5	0.02
Δ*E* · Δ*B (h/e*^2^)	6	0.5 × 10^−3^
