# Supplementary material for: Chern structure in the Bose-insulating phase of Sr2RuO4 nanofilms
Source: Sci Rep. 2017 Jan 23;7:41291. doi: 10.1038/srep41291 (PMC5256274; doi:10.1038/srep41291)
Supplement: Supplementary Information [file srep41291-s1.pdf]

Supplementary Information for 'Chern structure in the Bose-insulating phase of Sr_2RuO_4 nanofilms'

Hiroyoshi Nobukane,[1,2] Toyoki Matsuyama,[2,3] Satoshi Tanda[2,4]

[1]Department of Physics, Hokkaido University, Sapporo, 060-0810, Japan

[2]Center of Education and Research for Topological Science and Technology, Hokkaido University, Sapporo, 060-8628, Japan

[3]Department of Physics, Nara University of Education, Nara 630-8528, Japan

[4]Department of Applied Physics, Hokkaido University, Sapporo 060-8628, Japan

1. Electric transport properties in the insulating phase of Sr_2RuO_4 nanofilms

Figure S1(a) shows the temperature dependence of the Hall resistance R_{xy} and the longitudinal resistance R_{xx} for sample B with a thickness of 20 nm. With decreasing temperature, R_{xx} and R_{xy} increased with a log$T$ dependence, and exhibited flat tail resistance below 1.58 K. At a low temperature, the R_{xy} of 6.8 kΩ and the R_{xx} of 6.1 kΩ is close to the quantum resistance. The temperature of 1.58 K is consistent with the T_c in bulk Sr_2RuO_4. The differential conductance dI/dV_{xx} as a function of V_{xx} is shown in Fig. S1(b) for sample B and in Fig. S2 for sample A. We found superconducting gap structures above 1.5 K, although the non-zero resistivity of the flat tail was observed in Sr_2RuO_4 nanofilms.

2. Superconducting transition temperature in Sr_2RuO_4 single crystals with microscale thickness

Figure S3 shows the differential conductance dI/dV as a function of the bias current I for a Sr_2RuO_4 single crystal with a thickness of 400 nm (sample C), which is shifted vertically for clarity. A micrograph of sample C is shown in the left panel of Fig. S3. The tunneling spectra in the longitudinal electrode geometry were measured with a standard lock-in technique. We observed the superconducting gap structures below about 1.4 K. The microscale Sr_2RuO_4 sample grown with the solid phase reaction exhibited a T_c of about 1.5 K, although the T_c of Sr_2RuO_4 is suppressed by impurities [1]. Our samples with microscale

thickness show neither suppression of T_c nor enhancement to 3 K. Thus, we think that the samples are good quality single crystals. We also found conductance peaks in the vicinity of the zero bias current in the superconducting state. With decreasing temperature, the position of the conductance peak was shifted to a minus bias current below T_c. Our result reveals the existence of the gapless chiral Majorana state. The shift of the peak may be caused by the spontaneous chiral current.

[1] Mackenzie, A. P. *et al.* Extremely strong dependence of superconductivity on disorder in Sr_2RuO_4. *Phys. Rev. Lett.* **80**, 161 (1998).

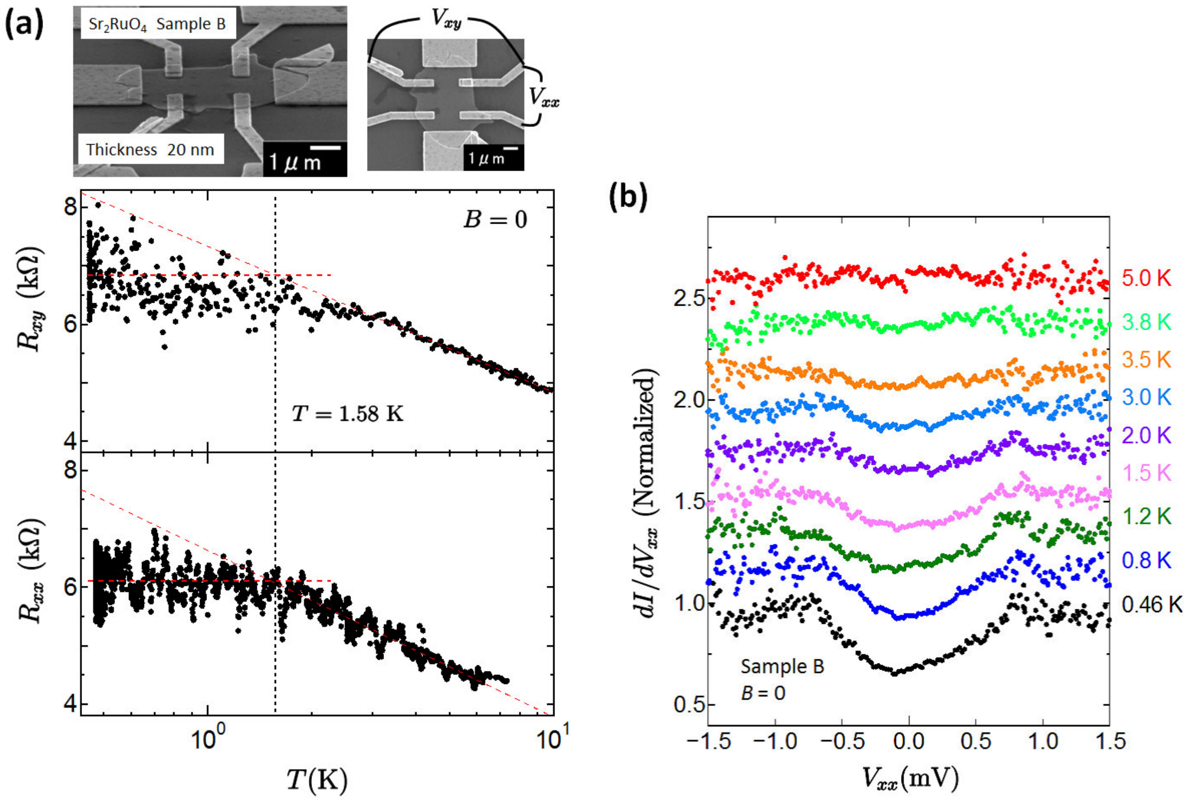

Supplementary Fig. S1: (a) Scanning electron micrographs of the top and side views of sample B with a thickness of 20 nm. Temperature dependence of R_{xy} and R_{xx} in a zero magnetic field. (b) The differential conductance dI/dV_{xx} as a function of V_{xx} at temperatures for sample B, which is shifted vertically for clarity.

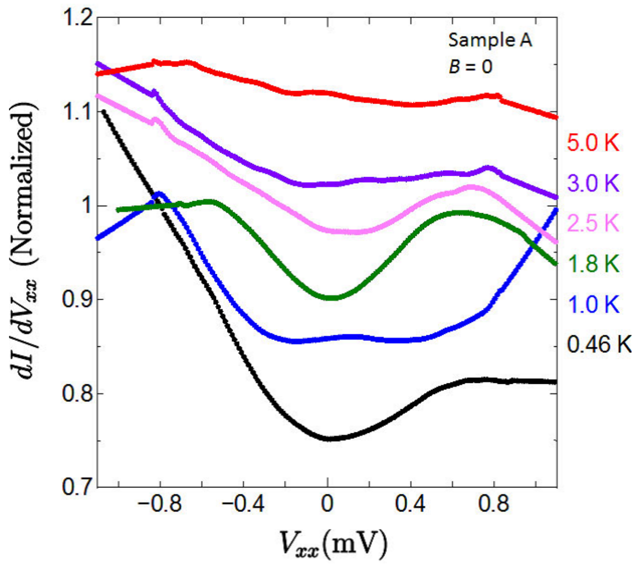

Supplementary Fig. S2: Differential conductance dI/dV_{xx} as a function of V_{xx} at several temperatures for sample A, which is shifted vertically for clarity.

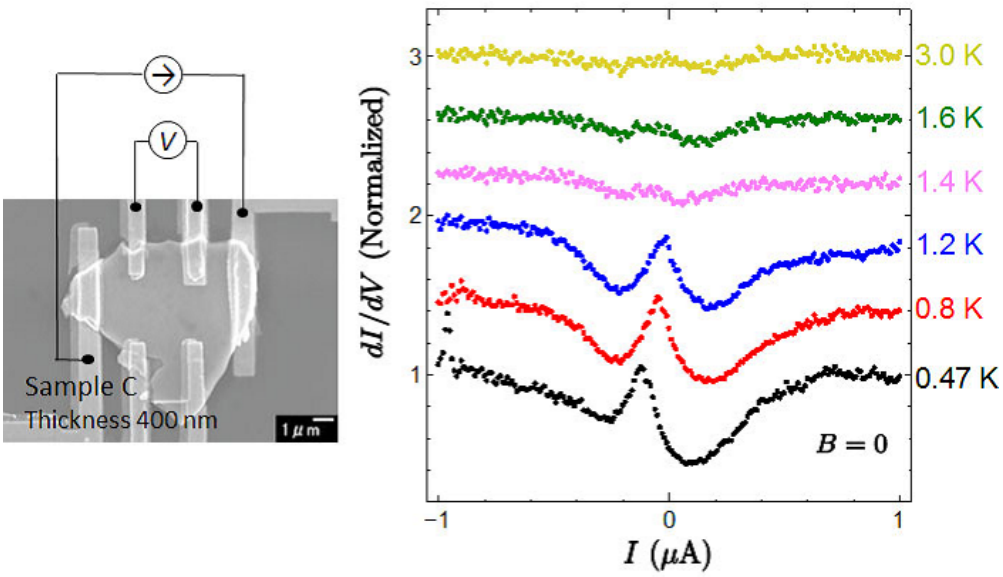

Supplementary Fig. S3: Differential conductance dI/dV versus bias current for several temperatures in the Sr_2RuO_4 single crystal with a thickness of 400 nm (sample C), which is shifted vertically for clarity. The zero bias conductance peak was found below 1.4 K.
